# Delayed Neurological Sequelae Successfully Treated with Adjuvant, Prolonged Hyperbaric Oxygen Therapy: Review and Case Report

**DOI:** 10.3390/ijerph19095300

**Published:** 2022-04-27

**Authors:** Luca Martani, Andrea Giovanniello, Gerardo Bosco, Luca Cantadori, Francesca Calissi, Dany Furfaro, Massimo Pedrazzini, Rosanna Vaschetto, Enrico Mario Camporesi, Matteo Paganini

**Affiliations:** 1 Vaio Hospital, 43036 Fidenza, Italy; lmartani@ausl.pr.it (L.M.); lcantadori@ausl.pr.it (L.C.); mpedrazzini@ausl.pr.it (M.P.); 2 Casa di Cura HABILITA I Cedri, 28073 Fara Novarese, Italy; andre.giova@gmail.com; 3 Department of Biomedical Sciences, University of Padova, 35131 Padova, Italy; matteo.paganini@unipd.it; 4 Rivoli Hospital, 10098 Rivoli, Italy; francesca.calissi@gmail.com; 5 Ospedale Parini, 11100 Aosta, Italy; dfurfaro@ausl.vda.it; 6 Department of Translational Sciences, University of Eastern Piedmont, 28100 Novara, Italy; rosanna.vaschetto@med.uniupo.it; 7 Department of Surgery, University of South Florida, Tampa, FL 33606, USA; ecampore@usf.edu

**Keywords:** carbon monoxide, hyperbaric oxygen, hyperbaric oxygen therapy, delayed neurologic sequelae, intoxication, poisoning

## Abstract

Carbon Monoxide (CO) intoxication is still a leading cause of mortality and morbidity in many countries. Due to the problematic detection in the environment and subtle symptoms, CO intoxication usually goes unrecognized, and both normobaric and hyperbaric oxygen (HBO) treatments are frequently administered with delay. Current knowledge is mainly focused on acute intoxication, while Delayed Neurological Sequelae (DNS) are neglected, especially their treatment. This work details the cases of two patients presenting a few weeks after CO intoxication with severe neurological impairment and a characteristic diffused demyelination at the brain magnetic resonance imaging, posing the diagnosis of DNS. After prolonged treatment with hyperbaric oxygen, combined with intravenous corticosteroids and rehabilitation, the clinical and radiological features of DNS disappeared, and the patients’ neurological status returned to normal. Such rare cases should reinforce a thorough clinical follow-up for CO intoxication victims and promote high-quality studies.

## 1. Introduction

Carbon Monoxide (CO) intoxication remains a leading cause of mortality and morbidity in many countries, mainly due to the difficult detection of this odorless, colorless, and tasteless gas in the environment. Because of its non-specific and subtle symptoms, CO poisoning is often missed by clinicians and underreported, hampering the accurate quantification of this threat. In the USA, a revision of the Emergency Department (ED) visits helped to obtain an incidence of about 50,000 cases per year [1], and the fatal cases considerably exceed 1000 deaths/year [2].

Symptoms of acute CO poisoning are not correlated with carboxyhemoglobin (CO-Hb) levels and can vary from malaise, nausea, vomiting, dizziness, and headache to loss of consciousness, seizures, and coma in most severe cases [3]. Moreover, various disturbances can appear and persist after the intoxication, primarily affecting areas with high metabolic rates. In fact, CO binds to other heme-containing cellular proteins—such as myoglobin in heart and skeletal muscle, cytochrome c oxidase in mitochondria, or platelet surface hemoproteins—severely impairing the aerobic metabolism and activating platelets. As a consequence, the equilibrium in tissues shifts towards inflammation and local damage [3].

In particular, Delayed Neurological Sequelae (DNS) are long-lasting neurological impairments that can occur even 2–6 weeks after the intoxication [4,5]. Cognitive impairment is often associated with neurological focal symptoms and depression/anxiety in these patients, potentially persisting years after the accidents [6].

A timely diagnosis is fundamental to early administrate high oxygen concentrations and prevent exacerbation of symptoms in CO poisoning. While Normobaric Oxygen (NBO) is the first-line treatment and the most readily available form of oxygen in standard care, current literature points towards Hyperbaric Oxygen (HBO) treatment as more effective in preventing and treating DNS [7]. HBO therapy promotes a faster removal of CO from hemoglobin in the blood and tissues by increasing oxygen partial pressure, counteracting inflammation, and mitochondrial dysfunction [8].

Unfortunately, DNS incidence and treatment are a neglected topic. For example, a recent case review conducted by some of the authors detailed the epidemiology and treatment modalities of 1383 patients treated in Italian hyperbaric chambers in the years 2015–2016. Only 12 patients with DNS were reported upon admission, and the outcome was difficult to assess since all were lost to follow-up [9]. The diagnosis of DNS is also difficult due to lucid intervals —frequently delaying the appearance of neurological symptoms. Also, other conditions can mimic the presentation of DNS, such as psychiatric conditions, other poisonings, or stroke. Recent studies confirm that a low Glasgow Coma Scale (GCS) at the first clinical encounter is associated with the occurrence of DNS, so this could be a useful predictor and trigger of a thorough clinical workup in cases of CO intoxication [10]. Other biochemical markers of brain damage, such as S-100 beta protein and neuron-specific enolase, have been studied but are not helpful in DNS detection [11]. The use of a neuropsychological battery of tests for the diagnosis of DNS have been proposed by Messier and Myers [12]. Moreover, Yang and coll. published a promising tool to predict DNS, but the use should be studied prospectively [13].

This work presents two cases of DNS that occurred in Northern Italy after CO poisoning and details long-term management and their outcomes.

## 2. Case Reports

### 2.1. First Case

In November 2018, a 42-year-old male was found unconscious by the firefighters in his house near Aosta (Northern Italy) after his relatives had not heard anything from him for two days. The patient was found on the ground, lethargic, with sphincter release, clearly dehydrated. His past medical history was unremarkable; he lived alone and was autonomous in daily living activities.

The patient was transported by the Emergency Medical Service (EMS) to the nearest ED without oxygen. The physical examination confirmed a GCS of 14 (E 4; V 4; M 6) due to confused answers and episodes of transient poor cooperation but without focal neurologic deficits or anomalies in the other systems. No obvious signs of trauma were detected at the physical exam.

**Figure 1:**
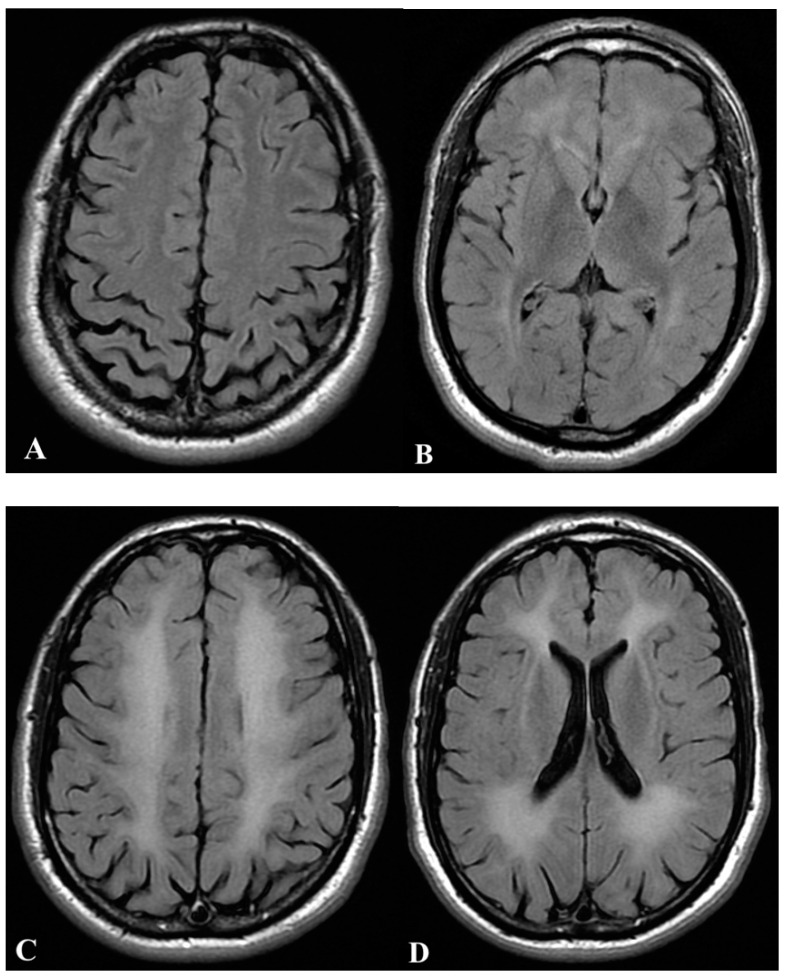
Brain Magnetic Resonance (MRI) two weeks after the carbon monoxide intoxication (**A**,**B**) showed no abnormalities; brain MRI after the development of neurological syndrome (**C**,**D**) showed bilateral and extended alteration of the supratentorial, periventricular, and centrum semiovale white matter, with hyper-intensity in Fluid-attenuated inversion recovery (FLAIR).

Vitals didn’t show any alteration, except for fever (38.0 °C), and finger stick glucose returned normal values. Brain and spine computed tomography (CT) and chest X-ray-showed no abnormalities. Once the common causes of altered level of consciousness were excluded, an accidental CO poisoning was suspected and confirmed by detecting 40.5% CO-Hb level [normal range: 0–2.0%] on venous blood gas analysis. The duration of exposure was unknown, and 100% oxygen was administered through a non-rebreather mask; after 60 min, CO-Hb dropped to 24.5%. Besides, ECG was normal, but high-sensibility T-troponin reached 36.92 ng/L (normal value: <13.99 ng/L). The overall case prompted a teleconsultation with a hyperbaric medicine physician. The patient was transferred to the nearest hyperbaric chamber in Fara Novarese (Novara, Italy) to undergo two sessions of HBO of 1 h each at 2.5 Atmospheres Absolute (ATA). After these two treatments, the patient was fully cooperative (GCS 15) but needed a third HBO session to treat persisting dizziness and bilateral thighs paresthesias. Troponin I values reached 157.60 ng/L (normal value: <38 ng/L) after the first treatment; after the HBO sessions, troponin I values dropped, and a transthoracic cardiac ultrasound did not show any abnormality in cardiac function. Due to the prolonged exposure to the toxicant and the high CO-Hb levels, the patient was screened for DNS with a neurological consult, and the patient was safely discharged home. A brain Magnetic Resonance Imaging (MRI) performed two weeks later showed no abnormalities (Figure 1A,B).

Unfortunately, three weeks after the event, the patient re-accessed the ED in Aosta, suffering from cognitive deterioration with scarce verbal expression and comprehension, postural instability, urinary and bowel incontinence, and ideomotor apraxia. After a second brain CT scan resulting normal, the patient was admitted to the neurology ward. Electroencephalography (EEG) detected nonspecific slow activity. At the same time, a new brain MRI revealed bilateral and extended alterations in the supratentorial, periventricular, and centrum semiovale white matter, accompanied by hyperintensity in Fluid-Attenuated Inversion Recovery (FLAIR) sequences (Figure 1C,D). Such important demyelination accompanied DNS onset as a late complication of CO poisoning. The patient was again transferred to the hyperbaric chamber of Fara Novarese and treated with a total of 21 HBO sessions of 1 h each at 2.5 ATA.

Additionally, intravenous methylprednisolone 500 mg per day was administered for 3 days, and the dose gradually reduced until discontinuation, along with intravenous N-acetylcysteine 300 mg/kg per day for 13 days, and speech and physical therapy (Supplementary Materials File S1). The patient progressively improved and recovered from his neurologic deficits, returning to his baseline status and resuming his normal daily routine. Further 10 HBO sessions were administered to strengthen the good results obtained. No adverse reactions to the drugs or HBO were reported at the end of the follow-up.

### 2.2. Second Case

In November 2019, a 68-year-old male and his wife were accidentally intoxicated by CO generated by a malfunctioning boiler. His wife found the patient in a catatonic state with a GCS of 9 (E 3; V 2; M 4), manifesting hyperthermia and urine incontinence, and was brought to the ED without oxygen. An arterial blood gas analysis showed CO-Hb values of 33.5% (range: 0–1.5%). The duration of exposure was unknown, and the patient was immediately provided with 100% oxygen through a non-rebreather mask. The ECG showed no pathological alterations, despite a T-troponin elevation detected (474 µg/L).

After 5 h, the patient reached the hyperbaric facility in Fidenza (Parma, Italy) and started the first HBO treatment at 2.5 ATA for 85 min. His wife was treated as well because of mild confusion. After the treatment, the patient’s neurological symptoms improved, and CO-Hb dropped to 0.4%, but a residual level of sedation was still detectable (GCS 14: E 4; V 4; M 6). The patient was transferred back and admitted to the high-acuity unit in the ED, continuing NBO for six hours. After achieving normal neurologic conditions and GCS 15, the patient was admitted to the Internal Medicine ward for five days due to a concomitant systemic infection caused by Enterococcus faecalis. T-troponin went down to average values and cardiac ultrasound did not show any abnormality in cardiac function.

**Figure 2:**
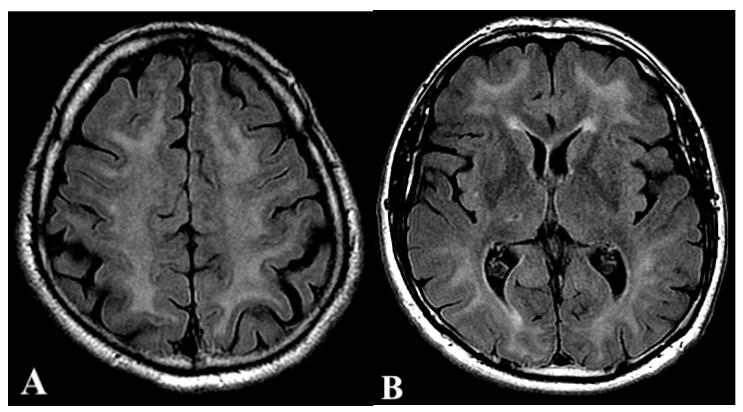

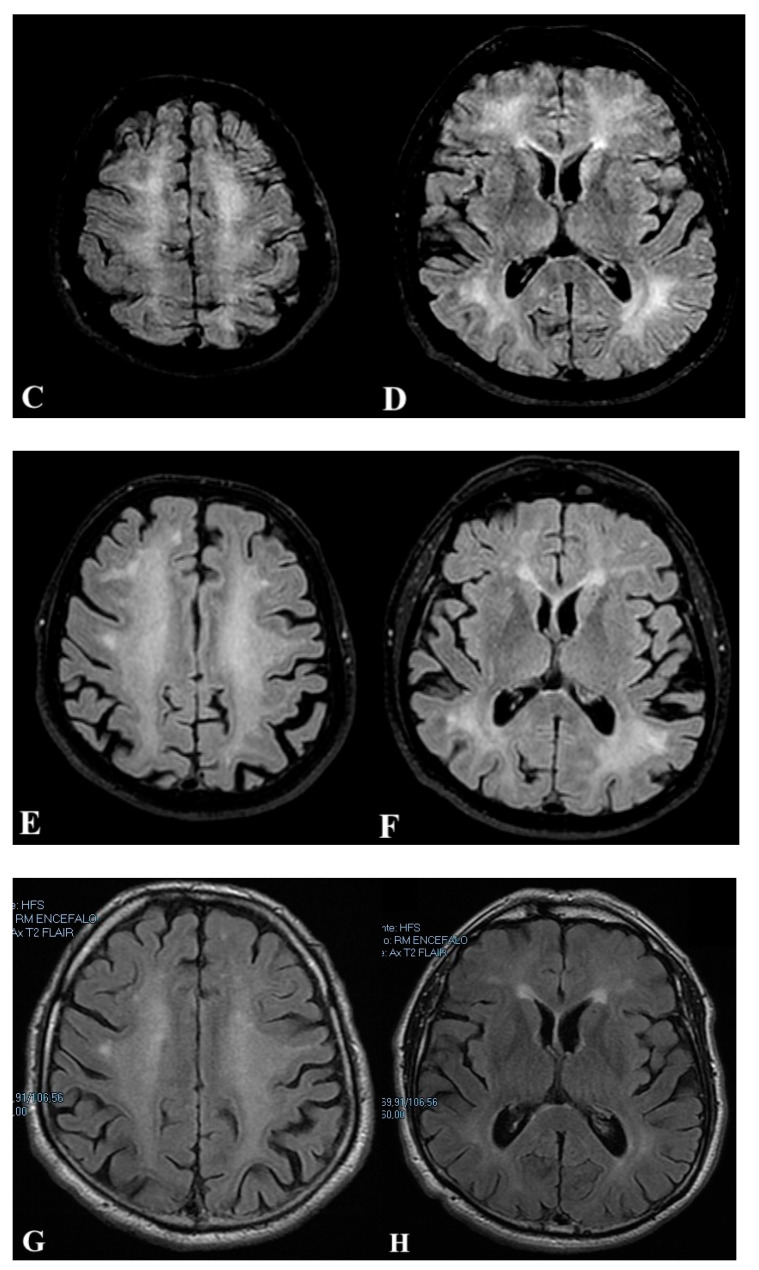
Brain Magnetic Resonance (MRI) 25 days after the carbon monoxide intoxication (**A**,**B**) showing diffused leukoencephalopathy; MRI after 8 hyperbaric oxygen (HBO) treatments with worsening findings (**C**,**D**); MRI after 36 days of HBO treatments showing persisting pathological findings (**E**,**F**); MRI performed 317 days after CO intoxication with general improvement (**G**,**H**). Sequences are fluid-attenuated inversion recovery (FLAIR).

Two weeks later, the patient re-accessed the ED due to progressive neurological impairment in the last three days, described by his wife as confusion, space and time disorientation, memory alterations, muscle stiffness, and progressive loss of coordination. A brain CT scan did not show any abnormality, and the patient was discharged after scheduling a brain MRI. Five days later, the patient returned because cognitive symptoms further worsened, and both bladder and bowel incontinence appeared. After being admitted to the Neurology ward, EEG showed slowed bilateral frontal signals with peaky aspects not modified by stimulations. The neurological examination revealed extreme stiffness of the whole body, small steps walking, bradylalia, hypo-amimia, and retropulsion. A first MRI showed diffused leukoencephalopathy (Figure 2A,B).

A series of 24 HBO daily sessions, 85 min each at 2.5 ATA, were coupled with daily physical medicine and rehabilitation sessions and 30 days of corticosteroids (intravenous dexamethasone 8 mg/day). A second brain MRI after 8 HBO treatments showed a generalized worsening of pathologic findings (Figure 2C,D). After 36 days of therapy, symptoms almost disappeared, leaving a severe cognitive deficit (Mini-Mental State Examination: MMSE = 8, 2/30; IADL = 1/8 (Self-Maintaining and Instrumental Activities of Daily Living) and ADL = 2/6 (Activities of Daily Living). A third brain MRI showed persistence of previous pathologic findings plus signal alterations in long-TR sequences at the cortical-subcortical junction (Figure 2E,F). The patient was then successfully discharged and transferred to a specialized center to undergo intensive physical rehabilitation for 24 days (Supplementary Materials S1). Except for a temporary re-hospitalization (5 days) due to pulmonary embolism and hypokalemia, the patient was finally sent home with a residual cognitive disorder. The last brain MRI performed 317 days after the intoxication showed a general improvement despite evidence of post-inflammatory cortical atrophy (Figure 2G,H). Due to the COVID-19 pandemic, the follow-up was resumed in October 2020. The patient demonstrated a surprising improvement in his cognitive domain (MMSE = 26, 2/30; IADL = 8/8; ADL = 6/6), and the patient resumed his sports activities (cycling). No adverse reactions to the drugs or HBO were reported at the end of the follow-up.

## 3. Discussion

The two presented cases manifested the deleterious neurologic consequences of CO intoxication days after the first presentation. In fact, after the stabilization of the clinical picture prompting a safe discharge, both patients re-accessed the ED with significant neurological deterioration. After a prolonged hospitalization combining pharmacological therapy, rehabilitation, and HBO treatments, the patients returned to an acceptable cognitive status, highlighting the success of multidisciplinary management of such complicated cases.

DNS manifests through neuropsychiatric symptoms with cognitive changes, sphincters incontinence, parkinsonism, akinetic mutism, and dystonia, occurring between 3 and 40 days after CO intoxication. The time between CO exposure and the development of the neuropsychiatric (or “lucid interval”) is essential to distinguish DNS from CO-induced acute brain damage or other potentially mimicking conditions. Together with symptoms and the proof of CO intoxication (detection of high CO levels in the environment or high CO-Hb % in the blood), the diagnosis of DNS is established with a brain MRI showing diffuse involving the white matter in both cerebral hemispheres. In both of these cases, it is essential to note that the second demyelination set of MRI, obtained 2 plus weeks after the first, showed worsening pathological findings, possibly indicating the slow delayed progression of cerebral pathology.

The incidence of DNS is still uncertain, ranging from 10–24% [14] to 46% [7], probably because the diagnosis is challenging and often underreported in medical records.

Further classifications of the long-term neurological effects of CO poisoning have been recently proposed. In DNS, there is a marked decline of at least one standard deviation on a specific psychological test score from a prior score, while in Persistent Neurological Sequelae (PNS), the cognitive impairment persists over six weeks after the event, as summarized by Ning et al. [15]. Marchinkowska et al., in their systematic review about the efficacy of HBO treatment in various neurological conditions with cognitive disturbances, combine PNS and DNS into DEACMP or “Delayed Encephalopathy after Acute CO Poisoning” [16]. However, the adoption of this term is still to be evaluated by scientific societies. A most recent paper by Kim et al. proposes two objective markers as pathognomonic of late neurological complications from CO intoxication, CPK, and MRI alterations [17]. Anyway, the authors believe that, due to the current uncertainty in the field, the term DNS and the need for delayed onset plus pathological neurologic picture and brain MRI alteration should still be used until a consensus is formally reached.

Overall, the exact pathogenesis of DNS remains unknown. The available literature mainly comprises case reports or series, and extensive randomized studies are unavailable. Since CNS tissues are most sensitive to oxygen deprivation, the cause of DNS has been primarily attributed to hypoxia. However, this theory does not explain all the clinical manifestations observed. Alternative physiopathological mechanisms have been proposed, such as immunological responses to chemically modified myelin components [18] or lipid peroxidation [19], leading to demyelination and leukoencephalopathy seen in MRIs [20]. Inflammation related to myeloperoxidase and reactive oxygen species can in fact elicit lymphocyte response, microglia activation, and lead to increased myelin basic protein levels on cerebrospinal fluid of those suffering from DNS [8]. This hypothesis could explain the effectiveness of medical agents such as combined corticosteroids and acetylcysteine (as administered in case No. 1) [21], N-butylphtalide, or dexamethasone [22]. Also, the use of HBO in treating acute CO poisoning and DNS is still controversial, and there is still not a standardized protocol. The Undersea and Hyperbaric Medicine Society (UHMS) recommends treating with HBO at a pressure from 2.5 to 3 ATA in case of neurologic symptoms, loss of consciousness, cardiovascular dysfunction, metabolic acidosis, or a 25% or higher CO-Hb level, even though this parameter poorly predicts the actual outcome of the patient [23]. The Italian Undersea and Hyperbaric Medicine Society (SIMSI) suggests the same protocol for CO intoxication [24], but both do not specifically address DNS. Nevertheless, HBO seems to decrease the risk of developing DNS [19] and the severity of neurological sequelae [25,26], but only with a large number of sessions (from 4 to over 40 treatments). Several adjuvant treatments such as antioxidant scavengers have been studied on animals [27], and showed promising results on humans. Specifically, N-acetylcysteine (NAC) is widely used as antioxidant in acetaminophen intoxication and provides the necessary substrates for glutathione production, counterbalancing the ROS produced by HBO; corticosteroids, as well, have a great potential in halting the inflammatory response and the immune system activation responsible of the intense demyelination seen on MRIs of DNS patients, and both act on reducing Nuclear Factor-kB activation [28]. Spina reported the successful concomitant use of NAC, corticosteroids, and a prolonged cycle of HBO in treating a young woman [28]. Xiang compared three groups of patients suffering from DEACMP and undergoing HBO therapy, treated without vs. two different regimes of IV dexamethasone, and reported a significantly higher improvement in the experimental group at 4 weeks, especially in the 10 mg/day vs. the 5 mg/day arm [29]. On the other hand, the use of a prolonged HBO treatment (100 sessions) without adjuvant drugs has been reported to be beneficial even 14 months after CO intoxication [30]; differently from the abovementioned cases, since prolonged HBO was administered months after the intoxication, the inflammatory damage was already established, and the use of corticosteroids would not have been beneficial. Therefore, more studies are still needed to solve the issue of adjuvant drugs in treating DNS. Also, on cardiac damage, b-blockers or melatonin have been tested on animal models of CO intoxication, but only as a pre-treatment and still need to be tested on humans. Specifically, the increase in biomarkers of cardiac damage without changes seen on ECGs at the admission and on cardiac ultrasound at the follow-up can be due to cell death without clinically significant ischemic lesions. Such explanation is consistent with previous findings of Fineschi et al. on human heart histological samples of fatal CO intoxications [31] and experimental CO intoxication and reoxygenation mouse models by Mizrak et al. [32].

These two cases represent a rare example of how DNS was successfully treated with a prolonged HBO cycle combined with corticosteroids and rehabilitation (speech and/or physical therapy, based on the symptoms). Nevertheless, the value of such treatments should be urgently validated by further high-quality studies. Among the other limitations of this paper, the MMSE scores were not retrieved for the first patient, and the rehabilitation therapies were different and not explicitly reported in the patients’ charts. Still, these features will be added to the ongoing Italian Registry of Carbon Monoxide Poisoning (IRCOP) [9].

## 4. Conclusions

These two cases of DNS show how CO poisoning is a challenging diagnosis and the delayed presentation of DNS with its typical lucid interval. The intensive treatment with prolonged HBO, medical therapy, and rehabilitation resulted in almost complete recovery in these two patients, suggesting the basis of a treatment protocol to be further tested and verified.

## Data Availability

Not applicable.

## Supplementary Materials

The following supporting information can be downloaded at: https://www.mdpi.com/article/10.3390/ijerph19095300/s1, File S1: Rehabilitation Protocols.
