# Supplementary material for: Delayed Neurological Sequelae Successfully Treated with Adjuvant, Prolonged Hyperbaric Oxygen Therapy: Review and Case Report
Source: Int J Environ Res Public Health. 2022 Apr 27;19(9):5300. doi: 10.3390/ijerph19095300 (PMC9104642; doi:10.3390/ijerph19095300)
Supplement: Supplementary file 1 [file ijerph-19-05300-s001.zip › ijerph-1650744-supplementary.pdf]

Supplementary Material File S1 – Rehabilitation targets

Case 1

The rehabilitation specialist evaluation found the patient as not able to walk autonomously, or to effectively perform daily life activities. Also, severe limb ataxia and scarce coordination were detected.

During the admission and then at home, the patient underwent the following physical therapy:

- limb movement in every direction
- coordination exercises (hands and feet)
- sitting to upright position and vice-versa
- stand-up position balance
- counteracting external stimuli of unbalance
- progressive autonomous walking

Case 2

The rehabilitation specialist found the patient as unable to walk autonomously, with unstable balance also when sitting in the bed, and with severe upper limb tremors.

During the admission and at home the patient underwent the following physical therapy:

- sitting to upright position and vice-versa
- rehabilitation to prolonged sitting or stand-up position
- balance in stand-up position
- progressive autonomous walking
